# Differentiated Thyroid Cancer Presenting With Solitary Bony Metastases to the Frontal Bone of the Skull

**DOI:** 10.7759/cureus.18735

**Published:** 2021-10-13

**Authors:** Christy M Moen, Richard B Townsley

**Affiliations:** 1 Ear, Nose and Throat (ENT), University Hospital Crosshouse, Glasgow, GBR; 2 Otolaryngology - Head and Neck Surgery, University Hospital Crosshouse, Kilmarnock, GBR

**Keywords:** cancer, otolaryngology, ent, thyroid cancer, oncology, metastasis, rare head and neck, diagnosis and management of bony lesions in maxillo facial region, bony metastasis

## Abstract

A 75-year-old lady was referred to the oral and maxillofacial surgery (OMFS) team with a painless swelling in the midline of her forehead. Investigations diagnosed it as a solitary metastasis of thyroid cancer. Follicular thyroid cancers are known to metastasise to bone; however, bony metastasis to the frontal bone of the skull is very rare. This case highlights how the effective use of a multidisciplinary team can lead to better patient outcomes. The patient went on to have a total thyroidectomy and received both radioactive iodine therapy and radiotherapy to the bony metastasis.

## Introduction

In this era of increasing medical complexity, patient outcomes are dependent on effective multidisciplinary teamwork. This report describes the management of a woman who had a very rare initial presentation of thyroid cancer. It required input from Primary Care, Oral and Maxillofacial Surgery (OMFS), Ear, Nose and Throat (ENT) surgeons, and Neurosurgery and Oncology clinicians.

Thyroid cancer is the most common endocrine malignancy, accounting for around 2.1% of all new malignancies worldwide [1]. Females are three times more likely to develop thyroid cancer than males, with incidence rates peaking between 40 and 44 years of age [2]. Thyroid cancer usually presents as a new mass in the neck region. Thyroid cancer presenting initially as a distant metastasis has been estimated at around 4% in previous studies [3]. It more commonly metastasises to lymph nodes, lungs and bones [4]. A solitary bony metastasis is a rare initial presentation of thyroid cancer, with a skull lesion being particularly rare [5].

## Case presentation

**Figure 1:**
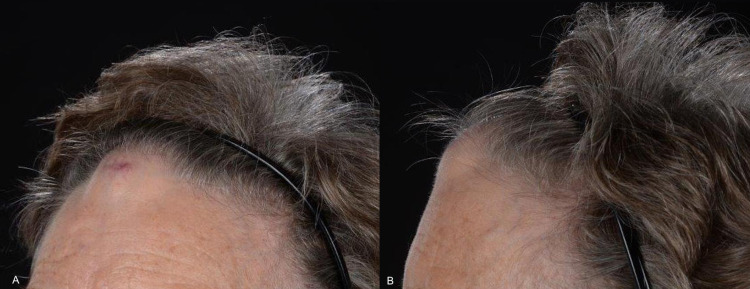
Photographs of the patient taken June 2020 (A, B).

The patient presented to her Primary Care Physician with a five-month history of an enlarging firm and non-tender forehead mass, around 4cm in diameter. There were no changes to the surrounding or overlying skin. There was no cervical lymphadenopathy and no obvious neck swelling. The patient was referred to the OMFS department at our institution (Figures 1A, 1B). Past medical history included aortic stenosis, previous coronary artery bypass graft, chronic kidney disease, chronic obstructive pulmonary disease, type 2 diabetes mellitus, hypertension and diverticulitis. Of note, the patient had Hodgkin’s lymphoma in 1974 which was treated with mantle radiotherapy.

**Figure 2:**
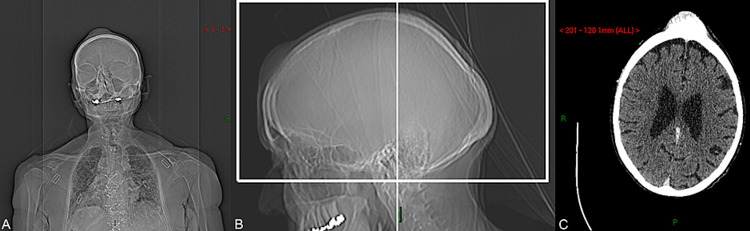
CT scan of patient in coronal (A), sagittal (B) and axial (C) views, respectively.

An incisional biopsy was taken of the lesion, and an urgent computerised tomography (CT) was requested. The results of the CT scan were in keeping with lymphoma; however, the biopsy confirmed metastatic thyroid cancer (histology report suggested papillary at the time) (Figures 2A-2C).

**Figure 3:**
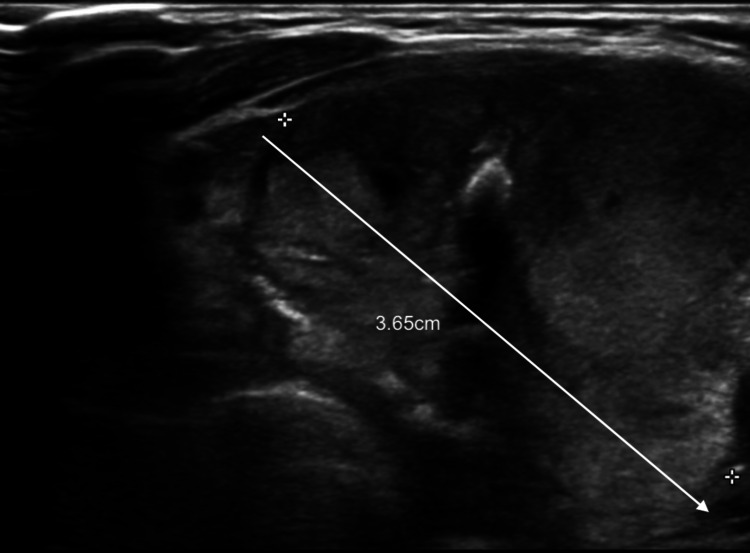
USS showing a 3.65cm U4 left thyroid nodule lesion (i.e., a suspicious thyroid nodule). USS - ultrasound

A CT head, neck, chest, abdomen and pelvis was requested, as well as an ultrasound (USS) scan of the neck. At this point, she was referred to the ENT team at University Hospital Crosshouse. The USS showed a 3.65cm U4 lesion in the isthmus of the thyroid propagating left into the left lobe, i.e., a suspicious thyroid nodule (Figure 3) [6].

**Figure 4:**
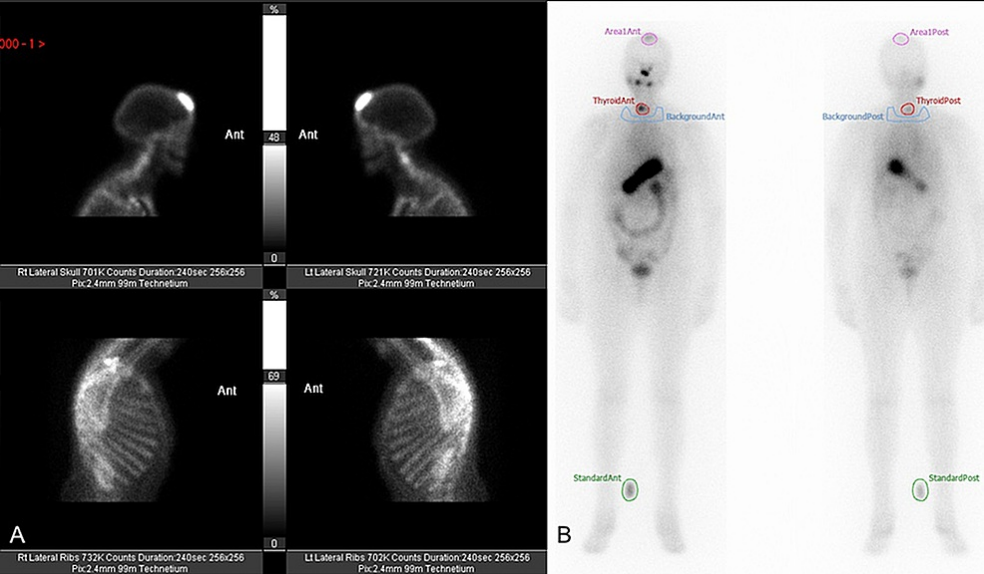
Nuclear medicine imaging showing areas of high uptake. (A) PET CT and (B) a nuclear medicine bone scan.

Fine needle aspiration was performed under USS guidance, which found thyroid follicular cells with atypia. No other metastasis was identified on Fluorodeoxyglucose-positron emission tomography. All scan results were in keeping with thyroid cancer and a solitary bony cranial vault metastasis (Figures 4A, 4B).

**Figure 5:**
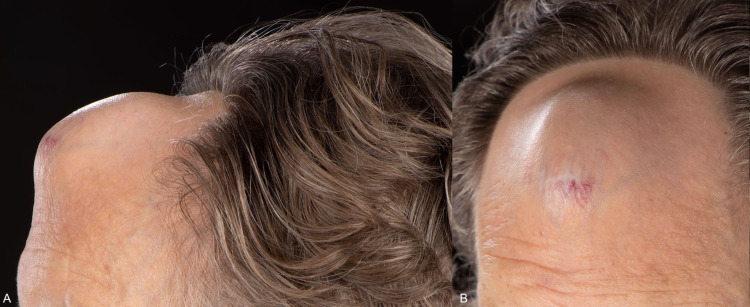
Photographs of the patient taken January 2021 (A, B).

The patient went on to have a thyroidectomy. The final pathology demonstrated a papillary carcinoma. The patient also received radioactive iodine with 3.7 Giga Becquerel with the goal of reducing the size of her bone lesion. As this did not have the desired effect, the patient went on to receive radical radiotherapy to the skull metastases at a dose of 55 Gray in 20 fractions. The patient receives regular follow up from both oncology and the Head & Neck team post-thyroidectomy (Figures 5A, 5B). It was decided by the MDT that resection of her bony lesion would not be in her best interest due to extensive comorbidities and depth of the lesion.

On a follow-up CT scan, it was found her bony disease had progressed and she also had new lung metastatic disease. The focus of the patient's treatment has now become palliative.

## Discussion

This is a rare case of metastatic thyroid cancer with metastasis to the frontal bone of the skull, picked up on a biopsy taken at an OMFS clinic. The initial differential diagnosis was an osteoma of the frontal bone. 

Thyroid cancer is relatively rare; however, it is the commonest endocrine cancer [7]. They typically present as a new neck lump; however, 60%-70% of patients with thyroid cancer will have a nonpalpable nodule that can be identified by imaging the thyroid gland with USS [1]. The majority of thyroid cancer is differentiated thyroid cancer (DTC), of which the variants papillary and follicular are most prevalent [8]. Most patients are euthyroid and asymptomatic.

Thyroid cancers are known to metastasise to bone. They most commonly metastasise to the spine, pelvis, sternum and ribs, extremities, shoulder girdle, and then craniomaxillofacial (5.4%) [9]. Nagamine et al. found 2.5% of thyroid cancers had metastasised to the skull [5]. Metastasis to the cranial vault is rare and rarer still are metastasis to the frontal bone. A literature review found only six cases of thyroid cancer that metastasised to the frontal bone, with two of those involving further bone sites. A review in 2018 found five cases of metastatic bony deposits to the frontal bone from a thyroid primary [10].

An important clinical principle to remember is that a bony metastasis from an unknown primary is more likely than a primary bone cancer [11].

The management of thyroid cancer begins with USS of any nodule or goitre [12]. Any suspicious lesion should have a fine-needle aspiration (FNA) biopsy taken for cytology. Cross-sectional imaging (CT or magnetic resonance imaging) should be undertaken in cases where the extrathyroidal spread is indicated.

Due to the incredibly small number of cases, there are no guidelines for the treatment of bony metastasis to the skull from a thyroid primary. The gold standard for the management of metastatic thyroid cancer involves total thyroidectomy. A decision is then made with regards to whether resection of the bony metastasis is in the best interests of the patient, with most patients having radioactive iodine therapy and radiotherapy.

## Conclusions

We have demonstrated an unusual case of metastatic thyroid cancer presenting as a solitary bony metastasis. Due to the rarity of these cases, it is important to discuss them at regional or national multidisciplinary teams to ensure the development of the experience and expertise of their management.

We believe there are some important learning points from this case. A bony metastasis from an unknown primary is more likely than primary bone cancer. Effective use of a multidisciplinary team allows for greater patient outcomes, and often the morbidity of a disease is more distressing than the mortality of the disease.
